# Current Evidence of a Deintensification Strategy for Patients with HPV-Related Oropharyngeal Cancer

**DOI:** 10.3390/cancers14163969

**Published:** 2022-08-17

**Authors:** Soo-Yoon Sung, Yeon-Sil Kim, Sung Hwan Kim, Seung Jae Lee, Sea-Won Lee, Yoo-Kang Kwak

**Affiliations:** 1 Department of Radiation Oncology, Eunpyeong St. Mary’s Hospital, College of Medicine, The Catholic University of Korea, Seoul 03312, Korea; 2 Department of Radiation Oncology, Seoul St. Mary’s Hospital, College of Medicine, The Catholic University of Korea, Seoul 06591, Korea; 3 Department of Radiation Oncology, St. Vincent’s Hospital, College of Medicine, The Catholic University of Korea, Seoul 16247, Korea; 4 Medical Library, The Catholic University of Korea, Seoul 06591, Korea; 5 Department of Radiation Oncology, Incheon St. Mary’s Hospital, College of Medicine, The Catholic University of Korea, Seoul 21431, Korea

**Keywords:** oropharyngeal cancer, human papillomavirus, p16, radiotherapy, chemotherapy, transoral surgery, deintensification, de-escalation

## Abstract

**Simple Summary:**

Human papillomavirus (HPV)-related oropharyngeal cancer represents a distinct disease entity, showing favorable treatment responses and survival outcomes. While the deintensification of treatment for HPV-related oropharyngeal cancer is widely considered necessary, details concerning patient selection and optimal strategies are undetermined. The heterogeneity of study populations and interventions in trials complicate the ability of physicians to apply the results in daily practice. The evolving landscape also requires physicians to consistently update the results of these trials. This article reviews the most recent evidence on the deintensification of HPV-related oropharyngeal cancer. We aim to provide physicians with some guidance regarding management options and assist researchers in appropriately designing trials in the future.

**Abstract:**

Human papillomavirus (HPV)-related oropharyngeal cancer differs from HPV-negative oropharyngeal cancer in terms of etiology, epidemiology, and prognosis. Younger and lower comorbidity patient demographics and favorable prognosis allow HPV-related oropharyngeal cancer patients to anticipate longer life expectancy. Reducing long-term toxicities has become an increasingly important issue. Treatment deintensification to reduce toxicities has been investigated in terms of many aspects, and the reduction of radiotherapy (RT) dose in definitive treatment, replacement of platinum-based chemotherapy with cetuximab, response-tailored dose prescription after induction chemotherapy, and reduction of adjuvant RT dose after transoral surgery have been evaluated. We performed a literature review of prospective trials of deintensification for HPV-related oropharyngeal cancer. In phase II trials, reduction of RT dose in definitive treatment showed comparable survival outcomes to historical results. Two phase III randomized trials reported inferior survival outcomes for cetuximab-based chemoradiation compared with cisplatin-based chemoradiation. In a randomized phase III trial investigating adjuvant RT, deintensified RT showed noninferior survival outcomes in patients without extranodal extension but worse survival in patients with extranodal extension. Optimal RT dosage and patient selection require confirmation in future studies. Although many phase II trials have reported promising outcomes, the results of phase III trials are needed to change the standard treatment. Since high-level evidence has not been established, current deintensification should only be performed as part of a clinical study with caution. Implementation in clinical practice should not be undertaken until evidence from phase III randomized trials is available.

## 1. Background for the Deintensification of HPV-Related Oropharyngeal Cancer

Oropharyngeal cancer accounts for 0.5% of all solid malignancies, and the incidence of oropharyngeal cancer has increased during the past three decades in the United States (US) [1,2]. A study investigating all 50 states of the US reported that oropharyngeal cancer increased by an average of 2.7% per year for men and 0.5% for women from 2001 to 2017 [3]. The rise in the incidence of oropharyngeal cancer is mainly due to an increase in human papillomavirus (HPV) prevalence and HPV-related oropharyngeal cancer [1,4]. HPV-related oropharyngeal cancer is a discrete disease entity distinguished from tobacco/alcohol-related oropharyngeal cancer; the clinical presentations and prognoses are different between these two conditions [5]. Patients with HPV-related oropharyngeal cancer present small primary tumors, advanced lymph node status, and have good prognoses after treatment.

Currently, the treatment for oropharyngeal cancer is the same regardless of the status of HPV. T1-2N0 disease is treated with a single modality, either surgery or radiotherapy (RT) alone. Locally advanced disease is usually treated with CRT or surgery followed by adjuvant RT/CRT [6,7]. For definitive treatment, the standard recommended RT dose administered to gross lesions is 70 Gy. For adjuvant treatment, 60 Gy is usually recommended for high-risk areas, and 63–66 Gy is recommended in cases of positive surgical margins or extranodal extension (ENE). Platinum-based concurrent chemotherapy is added as a radiosensitizer for the definitive treatment of locally advanced disease and adjuvant treatment of positive surgical margin or ENE [8,9,10]. The current treatment is effective in curing cancer but is accompanied by considerable acute and long-term toxicities. The adoption of IMRT decreased RT-related toxicities in head and neck cancer; however, dysphasia, xerostomia, and neck fibrosis commonly develop after this treatment [11,12]. With longer life expectancy, long-term toxicities and post-treatment QOL are more concerning.

**Figure 1:**
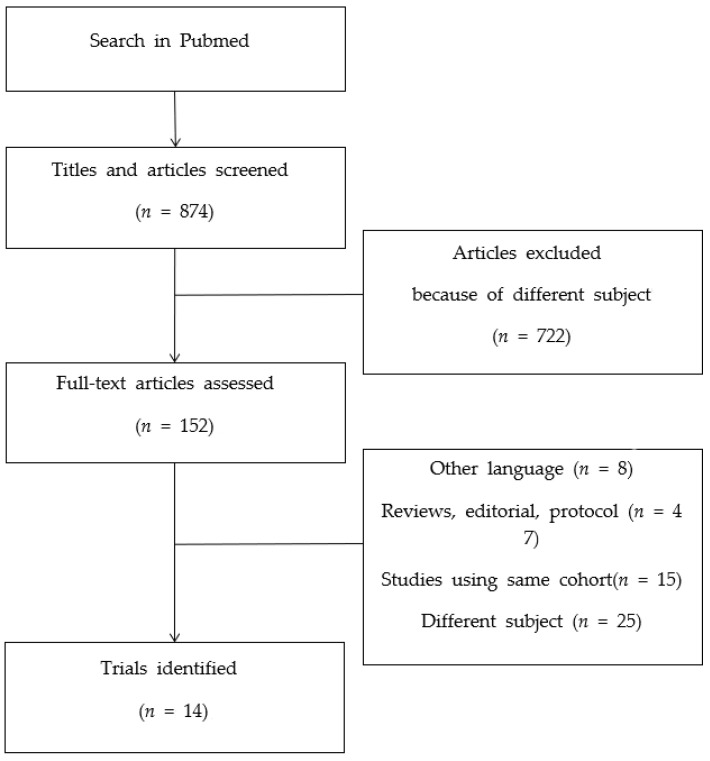
Flow diagram of the literature search of deintensification trials.

As HPV-related oropharyngeal cancer shows a favorable prognosis [5], attempts to deintensify treatment have been made to reduce toxicities without compromising survival outcomes. Various deintensification strategies have been investigated in clinical trials: RT dose reduction, patient stratification by clinical features, dose modification according to response to induction chemotherapy, replacement of platinum-based chemotherapy with cetuximab, and replacement of CRT with transoral surgery. Here, we carried out a literature review of prospective trials of deintensification for HPV-related oropharyngeal cancer and evaluated the current status of deintensified treatment for HPV-related oropharyngeal cancer. The PubMed and Clinicaltrials.gov databases were searched for relevant articles. A combination of the following search terms was used: “oropharyngeal cancer”, “oropharyngeal neoplasm”, “HPV”, “papillomavirus infection”, “papillomaviridae”, “p16”, “cyclin-dependent kinase inhibitor p16”, and “radiotherapy”. Only publications written in English were selected (Figure 1).

## 2. Deintensification of Definitive CRT

### 2.1. Staging of HPV-Related Oropharyngeal Cancer

Despite a favorable prognosis, HPV-related oropharyngeal cancer patients are usually diagnosed at a later stage due to massive neck node involvement, according to the 7th edition of the American Joint Committee on Cancer (AJCC) staging [5]. The 8th edition of the AJCC was updated to reflect the discrete features and prognosis of HPV-related oropharyngeal cancer [13,14]. The T4 stage is no longer divided into T4a and T4b according to the 8th edition of the AJCC. N1 was defined as a single ipsilateral lymph node ≤3 cm in the 7th edition, but in the 8th edition N1 includes ipsilateral node involvement regardless of number, while N2 includes bilateral node involvement and N3 includes nodes >6 cm. The AJCC, 7th edition classified T1N0M0 as stage I, T2N0M0 as stage II, and T1-3N1M0 as stage III. The AJCC, 8th edition classifies T0-2N0-1 as stage I and T0-2N2/T3N0-2 as stage II. A patient with a T1-2 primary tumor and multiple ipsilateral lymph nodes was classified as stage IV by the AJCC, 7th edition but now is classified as stage I by the AJCC, 8th edition.

### 2.2. HPV Testing and Clinical Relevance

HPV-specific testing includes direct detection of HPV DNA by in situ hybridization (ISH) or polymerase chain reaction (PCR) and detection of mRNA of the viral oncogenes E6 and E7 [15]. Detection of mRNA of E6/E7 is the current gold standard for identifying HPV-related oropharyngeal cancer [16,17,18], but the method needs high sample quality and technical equipment. HPV E6/E7 oncoproteins induce retinoblastoma protein (pRb) degradation, leading to the overexpression of p16 protein. Expression of p16 represents the transcriptional activity of high-risk HPV and can be used as a surrogate marker. p16 expression can be detected using immunohistochemistry (IHC), which is inexpensive and convenient. IHC testing of p16 showed a strong correlation with HPV mRNA [19]. The ASCO guideline recommends p16 IHC as a surrogate marker in a tissue specimen when there is ≥70% nuclear and at least moderate cytoplasmic expression [15].

However, a discrepancy was shown between p16 and HPV positivity. There is a subgroup who present HPV+/p16− or HPV−/p16+. A meta-analysis of 25 published articles on head and neck cancer investigated the clinical relevance of p16 and HPV positivity [20]. The subgroup proportions were 35.6% for HPV+/p16+, 50.4% for HPV−/p16−, 6.7% for HPV−/p16+, and 7.3% for HPV+/p16−. Subgroup analysis for only oropharyngeal cancer demonstrated the superior OS of HPV+/p16+ compared to those of HPV−/p16− (relative risk (RR), 2.87), HPV−/p16+ (RR, 2.26) and HPV+/p16− (RR, 2.67). HPV−/p16+ showed an improved OS compared with HPV−/p16− (RR, 0.78) but HPV+/p16- did not. Another study assessed a large-scale cohort of oropharyngeal cancer patients [21]. Among the 709 patients, 27.1% were HPV DNA+/p16+, 61.5% were HPV DNA −/p16−, 5.2% were HPV DNA+/p16−, and 5.5% were HPV DNA−/p16+. Patients with HPV DNA+/p16+ showed improved OS compared with the other three subgroups (*p* ≤ 0.019). Principal component analysis showed that patients with HPV DNA+/p16− clustered together with those with HPV DNA−/p16−, showing similar OS. These results suggested the potential of a new biological subtype of oropharyngeal cancer. The distinct biological behavior may be associated with clinical outcome. Selecting real HPV-driven oropharyngeal cancer would be critical for successful deintensification treatment.

### 2.3. Dose Reduction of Radiotherapy in a Definitive Setting

Definitive RT/CRT is the standard treatment for oropharyngeal cancer; it has the advantages of organ preservation and good functional outcomes compared with surgery [22,23,24]. Early-stage cancers, such as T1-2N0M0, can be cured with RT alone [25], and more advanced-stage cancers require the addition of concurrent chemotherapy. However, high-dose irradiation of normal tissue is effective in eradicating cancer cells and can cause long-term toxicities, such as swallowing difficulty, dry mouth, trismus, neck fibrosis, and osteonecrosis [26,27,28,29].

Chera et al. conducted a single-arm prospective phase II trial evaluating deintensified CRT with a definitive aim [30]. Patients staged T0-3N0-2cM0 according to the AJCC, 7th edition were included. HPV-positive or p16-positive tumors were eligible by fluorescence in situ hybridization (FISH) or IHC. The RT dose was reduced from a conventional regimen of 70 Gy to a deintensified dose of 60 Gy. The concurrent weekly cisplatin dose was reduced to 30 mg/m^2^, which is a 40% reduction. The primary endpoint was the rate of pathologic complete response (CR), confirmed by surgical evaluation. Surgeons performed a directed biopsy of the primary site in patients with clinical CR and a transoral resection in patients with clinical residual disease. A total of 44 patients were enrolled, and the pathologic CR rate was 86%. The 3-year local control and OS were 100% and 95%, respectively. The feeding tube insertion rate was 39%, and the 1-year dependence rate was 0%. The patients reported excellent long-term QOL and good swallowing function. This trial was the first to suggest good survival outcomes and low toxicities for a deintensified regimen. However, planned surgical evaluation could contribute to tumor control, especially in patients with clinical residual disease.

Based on a previous study, Chera et al. conducted another phase II trial that did not involve mandatory surgical evaluation [31]. The same deintensified RT dose and chemotherapy regimen were applied, and treatment response was assessed using PET-CT. p16+ oropharyngeal cancers pathologically confirmed by IHC were eligible. Analysis of the 114 patients indicated that the 3-year PFS and OS rates were 85% and 95%, respectively. Patients with a smoking history of up to 30 pack-years were included in this study if they quit smoking in the previous 5 years, but smoking history was not correlated with recurrence. The feeding tube insertion rate was 34%, and the 1-year dependence rate was 1%. In both trials by Chera et al., long-term swallowing difficulty was rare, and the most severe toxic effect was dry mouth.

The ORATOR-2 trial is a prospective phase II randomized trial comparing definitive deintensified CRT and transoral surgery [32]. Patients were eligible as T1-2N0-2M0 according to the AJCC, 8th edition, which also represents T1-2N0-2 by the AJCC, 7th edition. The tumor could be considered HPV-related on the basis of positive p16 status, real-time polymerase chain reaction, or in situ hybridization. Smoking history was not included as one of the eligibility criteria and was only used for stratification. For the CRT arm, gross lesions were treated with an RT dose of 60 Gy, with concurrent weekly cisplatin (40 mg/m^2^) in cases of multiple nodes or nodes ≥ 3 cm. For the transoral surgery arm, deintensified adjuvant RT was given according to the pathologic features. The dose administered to the tumor bed and positive neck level was 50 Gy, and the dose administered to patients with positive margins or ENE was 60 Gy. The study planned to enroll 140 patients but was terminated early due to an unacceptable incidence of death in the transoral surgery arm. Analysis after a median 17-month follow-up showed a 2-year OS rate of 100% in the CRT arm and 89% in the transoral surgery arm. The 2-year PFS was 100% in the CRT arm and 84% in the TORS arm. Despite early termination of the trial, 60 Gy CRT again showed excellent 2-year survival outcomes.

Trials in a definitive setting reduced the RT dose administered to 60 Gy and showed good survival outcomes and low toxicity profiles (Table 1). Tumors with a large burden, including T4 and N3, were not included in these trials. The long-term swallowing difficulty and feeding tube dependence rates were low compared with those in previous trials. A study involving 2315 patients reported feeding tube dependence rates of 7% at 1 year and 3.7% at 2 years after standard CRT [33]. A previous study showed that every additional 10 Gy to the muscular structure increased the probability of dysphasia by 19% [11]. A dose reduction of 10 Gy in deintensified trials may meaningfully decrease toxic effects. However, determinative evidence is insufficient because of the lack of randomized trials and short follow-up times. Phase III randomized trials comparing directly standard doses and deintensified doses in definitive CRT are necessary to generalize these encouraging results.

### 2.4. Dose Reduction of Elective Nodal Irradiation in a Definitive Setting

A single-arm prospective phase II trial evaluated the reduction of the RT dose administered to the elective nodal area [35]. Patients with oral cavity, oropharynx, larynx, and hypopharynx cancers were included. HPV status was not used as an eligibility criterion, but p16 status was confirmed to stratify patients. Patients with T3-4aN0-1 or T1-4aN2a-b stage disease (according to the AJCC, 7th edition) were eligible. Patients with T1-2N1 oral cavity or tonsil cancers were excluded because they did not require elective contralateral neck irradiation. Patients with N2c or bilateral N3 disease were also excluded because most neck lymphatics were at high risk of tumor, not the elective area. Patients with T2N0 base of tongue cancer were included because they have a risk for bilateral neck disease and require elective bilateral irradiation. Gross lesions were treated with 70 Gy in 35 fractions, and the elective nodal area was treated with 36 Gy in 18 fractions, with weekly cisplatin at 35 mg/m^2^. A total of 54 patients were enrolled, and 31 patients (57%) had HPV+ disease. No patient underwent elective nodal failure, except for one patient, who received selective neck dissection after CRT due to persistent disease. Four patients with HPV-negative disease underwent recurrence: two in the lungs and two in the 70 Gy area. Grade 3 acute toxicities were favorable but did not show a dramatic decrease. Dysphagia was observed in 80% of patients, mucositis/stomatitis was observed in 41%, and xerostomia was observed in 13%.

A multicenter prospective randomized noninferiority phase III study was conducted to compare the deintensified dose and conventional dose administered to the elective nodal area [36]. Squamous cell carcinoma of the oral cavity, oropharynx, hypopharynx, larynx, or unknown primary origin was included. T1-2N0 were eligible if elective nodal irradiation was performed. Both HPV+ and HPV- cancers of the oropharynx were included. Oropharyngeal cancers were regarded as HPV-related when both p16 by IHC and HPV-PCR were positive. Pretreatment neck dissection and concurrent chemotherapy were allowed according to the institution’s policy. All gross lesions were treated with 70 Gy in 2 Gy fractions. Patients were randomly assigned the dose administered to the elective nodal area: the 40 Gy arm or the standard 50 Gy arm. A total of 193 patients were evaluable, and HPV+ tumors accounted for 20.5% of all tumors. Dysphagia at 6 months was observed less frequently in the 40 Gy arm than in the 50 Gy arm (3.8% vs. 20.8%), but this difference did not reach statistical significance in a longitudinal analysis. However, salivary gland toxicity ≥grade 1 showed a significant difference in favor of the 40 Gy arm. The odds ratio between the 40 Gy group and the 50 Gy group for having no salivary gland toxicity was 1.88 (95% CI 1.07 to 3.31). With a median follow-up of 34.2 months, the 2-year OS was similar in both groups (72% in the 40 Gy arm vs. 73% in the 50 Gy arm, *p* = 0.73). Of 17 patients who underwent regional recurrence, 3 patients (18%) had elective nodal recurrence (2 in the 40 Gy arm and 1 in the 50 Gy arm). Among all 193 patients, the elective nodal failure rate was 1.6%, which is acceptably low.

A retrospective study at the Memorial Sloan Kettering Center (MSKCC) investigated the efficacy of a lower dose of 30 Gy to the elective nodal area [37]. HPV-related oropharyngeal cancer patients receiving definitive CRT were included. For a total of 276 patients, T1-2 disease was presented by 64.5% and T3-4 disease by 31.5% (according to the AJCC, 8th edition). N0-1 disease was presented by 76.4% and N2-3 disease by 23.5%. All gross lesions were treated with 70 Gy in 35 fractions and elective nodal areas were treated with 30 Gy in 15 fractions. For node-positive neck, retropharyngeal, retrostyloid, and levels II–IV were regarded as elective nodal areas. For node-negative neck, only levels II–IV were regarded as elective nodal areas. Levels IB and V were omitted. After a median follow-up of 26 months, overall 24-month locoregional control was 97.0%. Eight patients underwent locoregional recurrence, but all recurred lesions were in gross disease areas before CRT.

Two prospective trials showed that the RT dose administered to the elective nodal area can be reduced safely, despite the inclusion of HPV-negative disease. Regarding the favorable prognosis of HPV-related oropharyngeal cancer, elective 36–40 Gy irradiation seems safe for HPV-related oropharyngeal cancer. The elective nodal dose of 30 Gy showed excellent microscopic tumor control in a retrospective study at MSKCC. The result should be confirmed in prospective trials. These trials also reported relatively higher rates of dysphasia than expected, despite a reduced dose of elective nodal irradiation. A possible explanation for this finding would be the standard dose irradiation of gross lesions. All patients received 70 Gy to eradicate the gross tumor, and the dose administered to the pharyngeal structure might not substantially decreased. Instead, salivary gland toxicities were improved. The parotid gland and submandibular gland are anatomically close to the neck lymphatics. It is possible that the dose administered to the salivary gland largely depends on the dose administered to the lymphatic chain. Deintensification trials that reduced the dose administered to gross lesions from 70 Gy to 60 Gy reported a decrease in the incidence of dysphasia but not in xerostomia. The relationship between the dose administered to each target area and toxicities should be confirmed in future trials.

### 2.5. Omission of Chemotherapy in a Definitive Setting

The NRG oncology HN002 trial evaluated RT dose deintensification with omission of concurrent chemotherapy [34]. It was a phase II randomized trial comparing CRT vs. accelerated RT without concurrent chemotherapy, applying a deintensified dose regimen in both arms. Patients with T1-2N1-2bM0 or T3N0-2bM0 disease (according to the AJCC, 7th edition) and ≤10 pack-year smoking history were enrolled. p16+ tumors by IHC were eligible. Those with matted, supraclavicular, and infraclavicular lymph nodes were excluded. The RT dose was 60 Gy in both groups. It was delivered in five fractions per week for the CRT arm and 6 fractions per week for the RT alone arm. Patients in the CRT arm were treated with concurrent weekly cisplatin at 40 mg/m^2^. With 292 evaluable patients, the 2-year PFS and OS did not show a difference between the two groups (PFS, 90.5% vs. 87.6%, *p* = 0.20; OS, 96.7% vs. 97.3%, *p* = 0.93). However, the 2-year locoregional failure rate was significantly higher in the RT alone group (3.3% in CRT vs. 9.5% in RT alone, *p* = 0.02). The most common pattern of first failure was distant metastasis in the CRT group (35.3%) but local failure in the RT alone group (41.7%). The grade 3–4 acute toxic effect was higher in the CRT group (79.6% vs. 52.4%, *p* < 0.001), but long-term swallowing function was not different (feeding tube dependence rate at 6 months after RT, 2.8% vs. 3.8%).

A retrospective study by Lu et al. compared definitive CRT and RT alone in 189 patients [38]. Of 971 oropharyngeal patients treated between 2000 and 2008, 244 patients had available formalin-fixed paraffin-embedded (FFPE) tissues for p16 immunohistochemistry staining and 189 patients showed p16 positivity. Median RT dose was 66 Gy, and cisplatin was the most commonly used chemotherapy agent among CRT-treated patients. For stage I-II patients (according to the AJCC, 8th edition), CRT showed a significantly improved OS compared with RT alone (85.8% vs. 73.1%, *p* = 0.05). Another retrospective study compared definitive CRT and RT alone using the National Cancer Database (NCDB) in the United States (US). Out of 2830 patients, 1525 had HPV-positive (53.9%) and 1305 had HPV-negative (46.1%) oropharyngeal cancer. Only stage T1-3N0 disease cases were included. CCRT showed a significant improvement in OS for both HPV-positive T3N0 (85.2% vs. 65.9%, *p* < 0.01) and HPV-negative T3N0 disease (59.3% vs. 27.4%, *p* < 0.01). However, CCRT did not improve OS for T1N0 and T2N0 disease, regardless of HPV status. Omission of chemotherapy strategy should be investigated carefully for selected patients, because the benefit of concurrent chemotherapy has been shown in many studies.

### 2.6. Replacement of Cisplatin with Cetuximab

Cisplatin is the most common chemotherapy regimen that is concurrently used with RT for head and neck cancer treatment. Cisplatin-based CRT has been proven to show excellent survival outcomes in many clinical trials [10,39]. However, cisplatin is related to toxicities, including myelosuppression, anorexia, dysphagia, renal injury, and hearing impairment [40]. IMC9815 reported the efficacy of cetuximab as a radiosensitizer with comparable survival outcomes and low toxicity profiles [41]. To overcome the toxic effects of cisplatin, two prospective phase III randomized noninferiority trials, RTOG 1016 and De-ESCALaTE, were conducted to compare cisplatin and cetuximab.

RTOG 1016 included HPV-related oropharyngeal cancer patients at stage III–IV (T1-2N2-3 or T3-4N0-3, according to the AJCC, 7th edition) [42]. p16+ tumors by IHC were eligible. Patients in the cetuximab arm received 400 mg/m^2^ cetuximab as the loading dose 1 week before RT and 250 mg/m^2^ weekly during RT. Those in the cisplatin arm received 100 mg/m^2^ for the 3-week schedule, totaling 200 mg/m^2^ during RT. RT was delivered at 70 Gy in 35 fractions, with an accelerated schedule of six fractions per week. With 805 evaluable patients, the 5-year OS and PFS were significantly worse in the cetuximab group than in the cisplatin group (5-year OS, 77.9% vs. 84.6%, *p* = 0.0163; 5-year PFS, 67.3% vs. 78.4%, *p* = 0.0002). The hazard ratio of locoregional failure was twice that in the cetuximab group (HR 2.05, 95% CI 1.35–3.10, *p* = 0.0005). Unexpectedly, the overall rates of acute toxicities ≥grade 3 and late toxicities were similar between the two arms. The feeding tube dependence rate at 1 year after RT was also not different (8.4% vs. 9.2%, *p* = 0.7946).

The De-ESCALaTE trial was conducted with a similar study design to that of RTOG 1016 [43]. However, the eligible criteria were slightly different. Patients staged T3-4N0 or T1-4N1-3 and with a smoking history of ≤10 pack-years were eligible. p16+ tumors by IHC were eligible. High-risk HPV DNA by ISH was also performed. The cetuximab dose was the same as RTOG 1016, but the cisplatin dose was 100 mg/m^2^ for the 3-week schedule, with a total of 300 mg/m^2^ during RT. In the cisplatin group, only 38% of patients received all three cycles of chemotherapy. The median total dose of cisplatin was 200 mg/m^2^. All eight cycles of cetuximab were administered to 79% of the cetuximab group. Despite low compliance with cisplatin, a significant difference in 2-year OS, locoregional recurrence, and distant metastasis was observed in favor of the cisplatin arm (2-year OS, 97.5% vs. 89.4%, *p* = 0.0012). Grade 3–5 acute and late toxicities were not different between the two groups.

Despite promising data for IMC9815, cetuximab failed to show noninferior outcomes in direct comparison with cisplatin. The primary endpoint of RTOG 1016 was the noninferiority of 5-year OS and that of the De-ESCALaTE trial was overall grade 3–5 toxicities. Regardless of the different primary endpoints, both trials showed inferior survival outcomes for cetuximab and similar toxicities compared with cisplatin. Regarding the high rates of locoregional recurrence in the HN002, RTOG 1016, and De-ESCALaTE trials, concurrent cisplatin would be essential in definitive treatment for locally advanced HPV-related oropharyngeal cancer, except for T1-2N0M0 disease. A stepwise reduction of the cisplatin dose rather than complete omission might be a better strategy that can be examined in future trials. Additionally, these results suggest that deintensified regimens should be tested in direct comparison with conventional treatment before application to routine clinical management [44,45]. Indirect comparisons between different trials should be interpreted with caution because of heterogeneity in patient characteristics.

## 3. Deintensification in Response to Induction Chemotherapy

Induction chemotherapy was not interpolated into the standard treatment of locally advanced head and neck cancers because it failed to show improvements in OS and PFS in previous trials [46,47,48,49,50]. Instead, the concept of using induction chemotherapy to select good responders to treatment has evolved [51,52]. Good responders to induction chemotherapy respond well to CRT and have a lower tumor burden after induction chemotherapy.

The E2399 trial stratified patients according to response after induction chemotherapy. Deintensification was not applied to E2399 [53]. Resectable stage III or IV squamous cell carcinomas of the larynx or oropharynx were eligible. All patients received two cycles of paclitaxel (175 mg/m^2^) and carboplatin (AUC = 6), and the response was assessed. Patients with CR or partial response (PR) received CRT at the conventional dose of 70 Gy with weekly paclitaxel (30 mg/m^2^). Patients with stable disease (SD) or progressive disease (PD) received upfront surgical resection. A post hoc study using data from the E2399 trial analyzed the effect of HPV status on clinical outcomes [54]. The study reported that HPV-positive tumors showed a higher response rate than HPV-negative tumors after induction chemotherapy (82% vs. 55%, *p* = 0.01) and after CRT (84% vs. 57%, *p* = 0.007) for the entire cohort. Among patients with oropharyngeal cancer, OS and PFS also significantly improved in HPV-positive tumors compared with HPV-negative tumors (OS, *p* = 0.004; PFS, *p* = 0.05, respectively, log-rank test). Patients with HPV-positive tumors had a 61% lower risk of death (HR = 0.39, *p* = 0.06) and a 62% lower risk of progression (HR = 0.38, *p* = 0.09) than patients with HPV-negative tumors after adjustment for performance status. Based on the results of E2399, a few trials have investigated deintensified RT according to response after induction chemotherapy.

The E1308 trial was a single-arm phase II trial that investigated deintensification [55]. It included patients with resectable T1-4aN1-2 and T3-4aN0 (according to the AJCC, 7th edition). p16+ by IHC or HPV16+ by ISH tumors were eligible. The induction chemotherapy regimen was three cycles of cisplatin (75 mg/m^2^), paclitaxel (90 mg/m^2^), and cetuximab (400 mg/m^2^ for the loading dose and 250 mg/m^2^ weekly). After induction chemotherapy, patients with primary site CR were treated with a reduced dose of 54 Gy in 27 fractions. Those with less than CR were treated with a conventional dose of 69.3 Gy in 33 fractions. All patients received concurrent weekly cetuximab during RT. Primary site CR occurred in 70% of patients, and nodal CR occurred in 58%. All three cycles of induction chemotherapy were administered to 96.2% of patients, but protocol violation in RT dose occurred in 16% of patients. With a median follow-up of 35.4 months, the 2-year PFS was 80%, and the 2-year OS was 94% for patients with primary site-CR. The 54 Gy radiation group had less frequent grade 3–4 acute toxicities and a low incidence of swallowing difficulties (swallowing difficulty, 40% vs. 89%, *p* = 0.011). Interestingly, among the 54 Gy RT dose arm, a smoking history of >10 pack-years significantly decreased the 2-year PFS (92% vs. 57%, *p* = 0.0014).

A randomized phase II trial by Chen et al. was designed on the basis of E2399 [56]. Patients with resectable T1-4aN1-2 and T3-4aN0 disease (stage III and IV, according to the AJCC, 7th edition) were eligible. p16+ tumors by IHC were eligible. The same regimen of induction chemotherapy (paclitaxel 175 mg/m^2^ and carboplatin AUC = 6) and concurrent chemotherapy (paclitaxel 30 mg/m^2^) as E2399 was used. At 2 weeks after the induction chemotherapy, clinical response was evaluated. Patients with CR (11%) or PR (43%) received 54 Gy in 27 fractions. Other patients with SD (45%) received 60 Gy in 30 fractions, which was also the deintensified RT dose. The authors applied a deintensified dose scheme to all patients to different degrees based on the promising results of previous deintensification trials. The 2-year locoregional control rate, PFS, and OS for all patients were 95%, 92%, and 98%, respectively. Grade 3–5 acute toxicities were observed in 39% of patients. At 2 years after RT, grade 3 mucosal–esophageal toxicity was not different between the two arms (*p* = 0.47). The feeding tube dependence rate at 6 months after RT was 0%.

The OPTIMA trial investigated deintensified treatment not only for patients with early-stage tumors but also those with a large tumor burden, such as T4, N3, and those with a smoking history of >10 pack-years [57]. p16+ tumors by IHC were acceptable for enrollment. Confirmatory testing by PCR or ISH were performed in all patients. Patients were stratified into two groups: low-risk (T1-3, N0-2b unless bulky N2b, and smoking history of ≤10 pack-years) and high-risk (T4, N2c-3, bulky N2b disease, and smoking history of >10 pack-years). All patients received three cycles of induction carboplatin (AUC = 6) and nab-paclitaxel (100 mg/m^2^). After induction chemotherapy, low-risk patients with a ≥50% response received 50 Gy RT on a once-daily schedule without concurrent chemotherapy. Low-risk patients with a ≥30% response or high-risk patients with a ≥50% response received 45 Gy CRT with TFHX (paclitaxel 100 mg/m^2^, 5-fluorouracil infusion 600 mg/m^2^, and hydroxyurea 500 mg orally, twice daily). The low-risk patients with a <30% response, high-risk patients with a <50% response, and any patients with progression received 75 Gy CRT with TFHX. For patients receiving CRT, RT was delivered at 1.5 Gy twice daily. After completion of RT/CRT, surgical evaluation with neck dissection was performed to confirm the pathologic responses for the deintensification arm. The overall response rate of induction chemotherapy was 89%, and deintensified RT was given to 82% of patients. Response rates were similar between the low-risk group and the high-risk group. The pCR rate was 90% for all patients and 92% for those receiving deintensified treatment. With a median follow-up of 29 months, the 2-year PFS was 95% in the low-risk group and 94% in the high-risk group (*p* = 0.66). The 2-year OS rates were 100% in the low-risk group and 97% in the high-risk group (*p* = 0.84). Grade 3+ toxicity after induction chemotherapy was observed in 37% of patients. After RT/CRT, acute grade 3+ mucositis and dermatitis were significantly lower with deintensified treatment (mucositis, 30% vs. 63% vs. 91%, *p* = 0.004; dermatitis, 0% vs. 20% vs. 55%, *p* < 0.001). The requirement for a feeding tube was significantly lower in the deintensified treatment (0% vs. 31% vs. 82%, *p* < 0.0001). Although the pCR rate was as high as 90%, the possibility should be considered that surgical evaluation after RT/CRT contributed to the locoregional control and survival outcomes.

The Quarterback trial was a phase III randomized trial comparing deintensified 56 Gy CRT with conventional 70 Gy CRT after induction chemotherapy [58]. This trial included nasopharynx, oropharynx, supraglottic larynx, hypopharynx, and unknown primary cancers if p16+ was confirmed by immunohistochemistry. p16+ tumors by IHC were acceptable for the start of induction chemotherapy, but PCR confirmation was mandatory before randomization. Stage III–IV cancers according to the AJCC, 7th edition (T1-4aN1-2 or T3-4aN0) were eligible. Active smokers or those with a smoking history of >20 pack-years were excluded. Induction chemotherapy was three cycles of modified TPF (docetaxel 75 mg/m^2^, cisplatin 100 mg/m^2^, and 5-FU 750 mg/m^2^). Patients with CR or PR were randomized into the conventional 70 Gy CRT arm or the deintensified 56 Gy CRT arm. Other patients were treated with conventional 70 Gy CRT. Concurrent chemotherapy with carboplatin was planned weekly (AUC = 1.5). The initial target for enrolment was 365 patients, but the trial was terminated early due to lack of financial support and slow enrollment. For 20 patients enrolled, all patients showed an overall response to induction chemotherapy (CR 16 and PR 4). With a relatively long follow-up period of a median of 56 months, both the 3-year PFS and OS were 87.5% for the conventional 70 Gy arm and 83.3% for the deintensified 56 Gy arm. Due to the small sample size, noninferiority was not proven.

Four prospective trials investigating deintensification in response to induction chemotherapy reported favorable survival outcomes (Table 2). Good responders to induction chemotherapy seemed to be successfully treated with 50–55 Gy. However, there are a few points to be considered. First, great heterogeneity exists in regimens of induction chemotherapy and concurrent chemotherapy. E1308 used concurrent cetuximab, but cetuximab was proven to be inferior to platinum-based chemotherapy in RTOG 1016 and De-ESCALaTE. OPTIMA used a concurrent THFX and RT schedule of 1.5 Gy bid fractions. The study design of nonstandard treatment may cause difficulty in applying the results to routine clinical management. Second, induction chemotherapy is associated with considerable rates of toxicities. For patients receiving deintensification, it is debatable whether induction chemotherapy causes less toxicity than a reduced RT dose of 15 Gy. Moreover, poor responders were more heavily treated than in standard treatments. Delay of definitive CRT is another problem, because induction chemotherapy has no benefit in terms of survival. Direct comparisons between response-based deintensification and standard treatment should be performed despite encouraging data from these trials.

## 4. Deintensification of Adjuvant RT/CRT after Surgical Resection

Surgical resection followed by adjuvant RT is an alternative treatment for locally advanced oropharyngeal cancer if the expected postoperative functional outcome is acceptable. Reducing the adjuvant RT dose is one of the deintensification strategies (Table 3). It has the advantage that treatment can be decided based on the pathologic features. The E3311 trial was a phase II randomized trial that compared a deintensified dose of postoperative RT with conventional postoperative RT after primary transoral surgery [59]. p16+ oropharyngeal cancer patients with T1-2 cancers and no matted nodes were included. All patients received surgical resection using TORS or TLM. According to the pathologic features, patients were divided into three groups: low-risk patients (pT1-T2 with negative margins or N0-N1 without ENE), intermediate-risk patients (pT1-T2 with close margins <3 mm, N1-N2 with ≤1 mm ENE, or four or more positive nodes), and high-risk patients (positive margins, >1 mm of ENE, or ≥5 metastatic lymph nodes). Patients with an intermediate risk were randomly assigned to two treatment arms: a deintensified 50 Gy arm or a conventional 60 Gy arm. Low-risk patients did not receive adjuvant RT, and high-risk patients received 66 Gy CRT with weekly cisplatin. A total of 359 patients were evaluable for analysis. With a median 35.2-month follow-up, the 3-year PFS rates were 96.9% for the low-risk group, 94.9% for the 50 Gy intermediate-risk group, 93.4% for the 60 Gy intermediate-risk group, and 90.7% for the high-risk group. Smoking history did not have a negative prognostic impact on 3-year PFS in this trial. Toxicities ≥grade 3 after transoral surgery were observed in 17.1% of patients. Toxicities ≥grade 3 after adjuvant RT/CRT were observed in 14% of the 50 Gy intermediate-risk group, 24% of the 60 Gy intermediate-risk group, and 61% of the high-risk group. A significant difference was shown between the 50 Gy intermediate-risk group and the 60 Gy intermediate-risk group (*p* = 0.03).

The MC1273 trial assessed the most aggressive deintensification of RT dose [60]. Patients with pathologic stage III–IV disease according to the AJCC, 7th edition (T1-4aN1-2 or T3-4aN0) were enrolled after curative margin-clearing surgery. p16+ tumors by IHC were eligible. Eligible patients had either ENE or one of the following risk factors: lymphovascular invasion, perineural invasion, involvement of two or more regional nodes, any node >3 cm, or ≥T3 primary tumor. Patients were stratified into two groups according to the presence of ENE. Patients without ENE received 30 Gy CRT with weekly docetaxel (15 mg/m^2^). RT was delivered in 1.5 Gy fractions bid a day for 2 weeks. Those with ENE received an additional simultaneous boost to the nodal level, with ENE totaling up to 36 Gy in 1.8 Gy fractions bid. With a median follow-up of 35.7 months, the 2-year LRC was 100% in the 30 Gy group and 93.0% in the 36 Gy group. Local recurrence occurred in patients who underwent multiple margin excisions to achieve clear margins during surgery due to large T4 tumors or endophytic tumors. The 2-year PFS and OS for all patients were 91.1% and 98.7%, respectively. Grade 2–3 toxicities were 11.4% before the start of RT but improved to 9.2% and 1.4% 1 year and 2 years after CRT, respectively. The authors presumed that this improvement was because of recovery from surgery.

Based on the results of MC1273, a phase III randomized trial (MC1675) was performed [61]. All patients received TORS and achieved a negative surgical margin. HPV-related oropharyngeal cancer confirmed by p16+ on IHC were eligible. Patients with T4 disease or who required more than two attempts to clear margins were excluded. Patients were randomly assigned to the deintensified CRT group and the conventional CRT group. Patient stratification, RT dose schedule, and concurrent chemotherapy of the deintensification group were the same as those of the MC1273 group. The conventional CRT group received a 60 Gy RT dose and weekly cisplatin (40 mg/m^2^). Grade 3+ acute toxicity at 3 months after RT, which was the primary endpoint, was 1.6% in the deintensification group vs. 7.1% in the conventional group (*p* = 0.058). Swallowing function at 1 month and patient-reported QOL at 3 months were superior in the deintensification group. However, for patients with positive ENE, the deintensified arm showed worse 2-year PFS than the conventional arm (78.9% vs. 96.2%). This was especially true for patients with positive ENE and N2 disease, as the difference was larger (PFS, 42.9% vs. 100%; LRC, 77.0% vs. 100%; and DMFS, 59.4% vs. 100%). Patients with negative ENE showed similar 2-year PFS between the two groups (97.6% in the deintensification arm vs. 93.3% in the conventional arm). Based on the results of MC1675, the careful selection of patients is essential in successful deintensification. Deintensification may compromise survival outcomes for patients with definite high-risk features, such as positive margins, ENE, and large nodes.

The ADEPT trial is a phase III randomized controlled trial that was conducted to investigate deintensification for patients with ENE positivity (NCT01687413) [62]. Patients with node positivity, ENE positivity, T1-4a, and clear margins after transoral resection were eligible. p16+ by IHC was mandatory. Patients were randomly assigned to the 60 Gy CRT group or the 60 Gy RT alone group. Patient recruitment for this study has been terminated, but, ultimately, 42 patients were enrolled. The 1-year disease-free survival was 100% in the RT alone group and 90.9% in the CRT group. The 2-year locoregional control was 96.3% in the RT alone group and 81.8% in the CRT group. Further data are expected.

PATHOS is another ongoing phase II/III randomized trial evaluating deintensified adjuvant RT after transoral resection (NCT02215265) [63]. p16+ by IHC and HPV+ by PCR or ISH were required for enrollment. Patients were divided into three groups according to pathologic features. Patients with intermediate risk (T3 tumor, N2a-b, perineural invasion, vascular invasion, or margin < 5 mm) were randomly assigned to the deintensified 50 Gy arm and standard 60 Gy arm. Patients with high risk (margin < 1 mm or ENE) were randomly assigned to the deintensified 60 Gy RT alone arm and the 60 Gy CRT arm. The PATHOS trial is still recruiting.

## 5. Recent Changes in Patient Characteristics and Deintensification

Since Chaturvedi et al. reported an increase in oropharyngeal cancer in individuals aged <60 years, younger age has been regarded as a disease characteristic [64]. However, recent evidence has revealed a shift in disease burden to older age. In an epidemic study investigating all 50 states in the US, it was found that the incidence among men increased over 3% annually in a cohort of subjects aged ≥65 years between 2001 and 2017 [3]. Men aged 45–64 years showed a plateau after 2014. Among men younger than 45 years, the incidence declined after 2008. Among women, incidence increased in a cohort aged 55 to 64 years at an average of 1.6% per year, but decreased in a younger cohort aged <45 years at an average of 1.0% per year.

Another epidemic study using the SEER cancer registry reported an incidence pattern in successive birth cohorts [65]. Incidence of oropharyngeal cancer showed a rapid increase in white men born during 1939 to 1955 (5.3% per 2-year birth rate) and moderate increase in white men born during 1955 to 1969 (1.7% per 2-year birth rate). From 1992 to 2015, oropharyngeal cancer incidence increased strongly in white men aged 55 to 64 years (4.9% per year), 65 to 74 years (4.0% per year), and 45 to 54 years (3.7% per year). According to an analysis using age–period–cohort projection methods, oropharyngeal cancer incidence was estimated to increase dramatically in older age (>65 years) and to be stable when younger (<54 years).

This shift in disease burden to older age can be attributed to aging of the birth cohort. Increasing incidence in the cohort born from 1939 to 1955 is now manifesting an exponential increase in oropharyngeal cancer in the older age group (>65 years). Moderation in high-risk sexual behavior and reduction in smoking in recent years may explain the stabilization in the younger age cohort [66]. Vaccination for HPV is part of an effort to decrease the incidence of oropharyngeal cancer, but only adolescents and young adults (aged < 26 years) were candidates. Modeling studies anticipate a consistent increase in the incidence of oropharyngeal cancer in the older age group for at least one to two decades [67,68,69].

Change in the age of patients might have an impact on the clinical outcome of oropharyngeal cancer. Deintensification treatments basically assumed a good prognosis for HPV-related oropharyngeal cancer patients. Older patients have higher probabilities of co-morbidity and friability. While elderly patients may benefit from the low-toxicity profile of deintensification treatments, the clinical implications remain unclear. Clinical trials should consider change in patient characteristics and clarify the optimal management that is to be expected for older patients.

## 6. Conclusions

The optimal treatment for HPV-related oropharyngeal cancer is under investigation in clinical trials. Comparable survival outcomes and improved toxicity profiles were reported in phase II trials evaluating RT dose reduction in a definitive setting in response to induction chemotherapy and in an adjuvant setting. However, patients with ENE showed inferior outcomes after deintensified adjuvant RT. Deintensification treatment should be administered with great caution to high-risk patients with T4, N3, and positive ENE disease. Cetuximab failed to replace cisplatin as a concurrent chemotherapy agent. Long-term follow-up data for these trials are awaited with interest because HPV-related oropharyngeal cancer has a tendency of late relapse compared with HPV-negative oropharyngeal cancer.

Additionally, the promising results of phase II trials are not sufficient without confirmation by phase III trials. Direct comparisons with standard treatments in trials are mandatory before a paradigm shift. These new paradigms could result in unexpected negative impacts on survival outcomes. As evidence from randomized phase III trials with long-term follow-ups have not been established, the deintensification strategy should only be performed as a part of a clinical study with caution [70,71]. Implementation in clinical practice should not be undertaken until high-level evidence is available. Furthermore, new deintensification strategies should be confirmed in phase II trials before proceeding to phase III trials in order to avoid possible detrimental effects on the survival of enrolled patients. Close monitoring and interim analysis are recommended for clinical trials investigating deintensification.

## Figures and Tables

**Table 1:** Deintensification trials in a definitive setting.

Study	Design	No. of Patients	Patient Eligibility ^1^	Intervention Arm/Outcome/Toxicity
*Dose reduction of definitive RT*				
Chera et al. [30] (NCT01530997)	Phase II Single arm	44	T0-3N0-2M0 HPV + (ISH) or p16+ (IHC) Smoking ≤ 10 PY or > 10 PY/abstinent for 5 years	**Arm** CRT (weekly cisplatin 30 mg/m^2^) followed by surgical evaluation High-risk region: 60 Gy/low-risk region: 54 Gy **Outcome** pCR rate: 86%/3-year LC: 100%/3-year OS: 95% **Toxicity** Feeding tube insertion: 39%/1-year dependence rate: 0%
Chera et al. [31] (NCT02281955)	Phase II Single arm	114	T0-3N0-2cM0 p16+ (IHC) Smoking ≤ 10 PY or ≤ 30 PY/abstinent for 5 years	**Arm** CRT (weekly cisplatin 30 mg/m^2^) High-risk region: 60 Gy/low-risk region: 54 Gy **Outcome** 3-year LC: 94%/3-year PFS: 85%/3-year OS: 95% **Toxicity** Feeding tube insertion: 34%/1-year dependence rate: 1%
*Chemotherapy omission with RT dose reduction*				
HN-002 [34] (NCT02254278)	Phase II Randomized	292	T1-2N1-2bM0/T3N0-2bM0 p16+ (IHC) Smoking history ≤ 10 PY	**Arm** CRT 60 Gy (weekly cisplatin 40 mg/m^2^) vs. RT alone 60 Gy **Outcome** 2-year PFS: 90.5% vs. 87.6% (*p* = 0.20) 2-year local failure: 3.3% vs. 9.5% (*p* = 0.02) **Toxicity** Grade 3–4 acute toxicity: 79.6% vs. 52.4% (*p* < 0.001) Feeding tube dependence at 6 months: 2.8% vs. 3.8%
*Dose-reduced RT vs. surgery*				
ORATOR-2 [32]	Phase II Randomized	61	T1-2N0-2 p16+ or HPV + (ISH, RT-PCR) Early termination due to excessive toxicity in TORS arm	**Arm** CRT 60 Gy (weekly cisplatin 40 mg/m^2^) vs. TORS +/− adjuvant RT 50 Gy **Outcome** Immature data with 17 months f/u 2-year OS: 100% vs. 89% 2-year PFS: 100% vs. 84% **Toxicity** Toxicity ≥ grade 2: 67% vs. 71%
*Dose reduction to elective nodal area*				
Maguire et al. [35]	Phase II Single arm	54	Oral cavity, oropharynx, larynx, hypopharynx cancer T3-4N0-1/T1-4N2a-b	**Arm** CRT 36 Gy to elective nodal area (weekly cisplatin 35 mg/m^2^) Gross lesion: 70 Gy/elective nodal area: 36 Gy **Outcome** Elective nodal failure 0% **Toxicity** Dysphagia 80%, mucositis/stomatitis 41%, xerostomia 13%
Nevens et al. [36]	Phase III Randomized	193	Oral cavity, oropharynx, larynx, hypopharynx, unknown primary cancer	**Arm** CRT 40 Gy vs. 50 Gy to elective nodal area Gross lesion: 70 Gy/chemotherapy: allowed **Outcome** 2-year OS 72% vs. 73% (*p* = 0.73) Elective nodal failure 2.1% vs. 1.0% **Toxicity** Dysphagia 80%, mucositis/stomatitis 41%, xerostomia 13%

Abbreviations: CRT, chemoradiotherapy; IHC, immunohistochemistry; ISH, in situ hybridization; LC, local control; OS, overall survival; pCR, pathologic complete remission; PFS, progression-free survival; PY, pack-year; RT, radiotherapy; RT-PCR, real-time polymerase chain reaction; TORS, transoral robotic surgery. ^1^ All stages were classified according to the AJCC, 7th edition.

**Table 2:** Deintensification in response to induction chemotherapy trials.

Study	Design	No. of Patients	Patient Eligibility ^1^	Intervention/Outcome/Toxicity
E1308 [55] (NCT01084083)	Phase II Stratification	80	T1-4aN1-2 or T3-4aN0 Resectable disease p16+ (IHC) or HPV16+ (ISH)	**Arm** IC followed by CRT IC: cisplatin, paclitaxel, cetuximab/concurrent chemotherapy: cetuximab 1. Primary site CR: 54 Gy 2. Primary site not CR: 69.3 Gy **Outcome** Primary site CR: 2-year PFS 80%/2-year OS 94% All patients: 2-year PFS 78%/2-year OS 91%. **Toxicity** Dysphagia at 2 year: 40% in ≤ 54 Gy vs. 89% in 69.3 Gy (*p* = 0.011)
Chen et al. [56] (NCT02048020 NCT01716195)	Phase II Stratification	44	T1-4aN1-2 or T3-4aN0 p16+ (IHC)	**Arm** IC followed by CRT IC: paclitaxel, carboplatin/Concurrent chemotherapy: paclitaxel 1. CR, PR: 54 Gy 2. SD: 60 Gy **Outcome** 2-year LRC: 95%/2-year PFS: 92%/2-year OS: 98% **Toxicity** Acute toxicity ≥ grade 3: 39% Feeding tube dependency at 6 months: 0%
OPTIMA [57] (NCT02258659)	Phase II Stratification	62	T1-4aN2-3 or T3-4Nany p16+ (IHC) Stratification: 1. Low-risk: T1-3, N0-2b, smoking ≤ 10 pack-year 2. High-risk: T4, N2c-3, bulky N2b, smoking > 10 pack-year	**Arm** IC followed by RT/CRT IC: carboplatin, nab-placlitaxel/concurrent chemotherapy: TFHX 1. 50 Gy RT alone: low-risk ≥ 50% response 2. 45 Gy CRT (BID): low-risk ≥ 30%, high-risk ≥ 50% 3. 75 Gy CRT (BID): low-risk < 30%, high-risk < 50%, any risk with PD **Outcome** 2-year PFS: low-risk 95%, high-risk 94% 2-year OS: low-risk 100%, high-risk 97% **Toxicity** Mucositis ≥ grade 3: 30% vs. 63% vs. 91% Dermatitis ≥ grade 3: 0% vs. 20% vs. 55% Feeding tube dependency: 0% vs. 31% vs. 82%
Quarterback trial [58] (NCT01706939)	Phase III Randomized	20 (Early termination)	T1-4aN1-2 or T3-4aN0 P16+ (IHC) and HPV+ (PCR) Oropharynx, nasopharynx, hypopharynx, larynx, unknown primary cancer Smoking history ≤ 20 PY	**Arm** IC + CRT 70 Gy vs. IC + CRT 56 Gy IC: modified TPF 3 cycles/concurrent chemotherapy: weekly carboplatin 1–2. CR, PR: randomized to two groups, 70 Gy CRT vs. 56 Gy CRT 3. SD, PD: 70 Gy CRT **Outcome** 3-year PFS: 70 Gy arm 87.5% vs. 56 Gy arm 83.3% 3-year OS: 70 Gy arm 87.5% vs. 56 Gy arm 83.3%

Abbreviations: BID, bis in die (twice a day); CR, complete remission; CRT, chemoradiotherapy; IC, induction chemotherapy; IHC, immunohistochemistry; ISH, in situ hybridization; LRC, locoregional control; OS, overall survival; PCR, polymerase chain reaction; PD, progressive disease; PFS, progression-free survival; PR, partial regression; PY, pack-year; RT, radiotherapy; SD, stable disease; TFHX, placlitaxel, 5-fluorouracil, hydroxyurea; TORS, transoral robotic surgery; TPF, docetaxel, cisplatin, and 5-fluorouracil. ^1^ All stages were classified according to the AJCC, 7th edition.

**Table 3:** Deintensification trials in the postoperative setting.

Study	Design	No. of Patients	Patients Eligibility ^1^	Intervention/Outcome/Toxicity
E3311 [59] (NCT01898494)	Phase II Randomized	359	T1-2 p16+ (IHC) No matted node	**Arm** Transoral sugery + observation/RT/CRT 1. Low risk: observation 2-3. Intermediate risk: randomization into 50 Gy vs. 60 Gy 4. High risk: 66 Gy CRT (weekly cisplatin 40 mg/m^2^) **Outcome** 3-year PFS: 96.9% vs. 94.9% vs. 93.4% vs. 90.7% 3-year OS: 100% vs. 99% vs. 98.1% vs. 96.3% **Toxicity** toxicity ≥grade 3 after CRT: none vs. 14% vs. 24% vs. 61% (*p* = 0.03)
MC1273 [60] (NCT01932697)	Phase II Stratification	79	Pathologic III–IV p16+ (IHC) With ENE or one of risk factor (LVI, PNI, ≥2 regional nodes, any node >3 cm, or ≥T3)	**Arm** Transoral surgery + CRT 1. ENE (−): 30 Gy CRT (weekly docetaxel 15 mg/m^2^) 2. ENE (+): 36 Gy CRT (weekly docetaxel 15 mg/m^2^) **Outcome** 2-year LRC: 100% vs. 93.0% 2-year overall PFS: 91.1%/2-year overall OS: 98.7%. **Toxicity** grade 2-3 toxicities: 11.4% before start of CRT 9.2% at 1 year after CRT, 1.4% at 2-year
MC1675 [61] (NCT02908477)	III randomized	194	p16+ (IHC) ≥1 of following risk factor: Number of LN ≥ 2, LN > 3 cm, PNI, LVI, T3, ENE	**Arm** surgery + deintensified RT vs. surgery + standard RT/CRT 1. ENE (−): 30 Gy CRT (docetaxel 15 mg/m^2^) vs. 60 Gy RT (cisplatin 40 mg/m^2^) 2. ENE (+): 36 Gy CRT (docetaxel 15 mg/m^2^) vs. 60 Gy CRT (cisplatin 40 mg/m^2^) **Outcome** ENE (−): 2-year PFS, 97.6% vs. 93.3% ENE (+): 2-year PFS, 78.9% versus 96.2% **Toxicity** acute toxicity ≥ grade 3 at 3 months: 1.6% vs. 7.1% (*p* = 0.058).
ADEPT [62] (NCT01687413)	III randomized	42	T1-4a, N+, positive ENE, clear margin p16+ (IHC)	**Arm** transoral resection + 60 Gy RT vs. transoral resection + 60 Gy CRT concurrent chemotherapy: weekly cisplatin 40 mg/m^2^ **Outcome** 1-year DFS: 100% vs. 90.9% 2-year LRC: 96.3% vs. 81.8%
PATHOS [63] (NCT02215265)	II/III randomized	recruiting	T1-3N0-2b p16+ (IHC) and HPV+ (PCR or ISH)	**Arm** Transoral surgery + observation/RT/CRT 1. Low risk: no adjuvant treatment 2–3. Intermediate risk: randomized into 50 Gy vs. 60 Gy 4–5. high risk: randomized into 60 Gy RT vs. 60 Gy CRT (cisplatin) **Outcome** primary endpoint: MDADI, OS

Abbreviations: CRT, chemoradiotherapy; DFS, disease-free survival; ENE, extranodal extension; IHC, immunohistochemistry; ISH, in situ hybridization; LRC, locoregional control; LVI, lymphovascular invasion; MDADI, MD Anderson Dysphagia Inventory; OS, overall survival; PCR, polymerase chain reaction; PFS, progression-free survival; PNI, perineural invasion; RT, radiotherapy; TORS, transoral robotic surgery. ^1^ All stages were classified according to the AJCC, 7th edition.
